# The diagnosis and treatment of testicular torsion in children with non-scrotal initial symptoms

**DOI:** 10.3389/fped.2023.1176345

**Published:** 2023-06-15

**Authors:** Chang-Kun Mao, Yong-Sheng Cao, Xiang Liu, Bo Peng, Han Chu, Qi-Fei Deng, Xin Yu, Cheng-Pin Tao, Tao Zhang, Chao Yang, Shan Peng

**Affiliations:** Department of Urology, Anhui Provincial Children’s Hospital, Hefei, China

**Keywords:** testicular torsion, misdiagnosis, orchiopexy, children, bell clapper deformity

## Abstract

**Objective:**

To explore the clinical characteristics of testicular torsion in children with non-scrotal initial symptoms who were misdiagnosed.

**Methods:**

A retrospective analysis of 73 cases children with testicular torsion and non-scrotal symptoms who were admitted to our department from October 2013 to December 2021 was performed. Patients were divided into misdiagnosis (27 cases) and clear diagnosis at first visit (46 cases) groups. Clinical data, including age at surgery, clinical presentation, physical examination, number of visits (≥2 times), affected side, time from initial symptoms to surgery, and surgical outcomes, were collected. The TWIST (Testicular Workup for Ischemia and Suspected Torsion) score was calculated and analyzed.

**Results:**

Statistically significant differences between the misdiagnosis and clear diagnosis groups were seen in the time from initial symptoms to surgery, the number of visits, the degree of testicular torsion, and the rate of orchiectomy (*P *< 0.05). There were no statistically significant differences (*P *> 0.05) in age, affected side, TWIST score, guardian, direction of testicular torsion, intra-vaginal or extra-vaginal torsion, and Arda classification. Postoperative follow-up was 6–40 months. Of the 36 patients who required an orchiopexy, 1 had testicular atrophy at six months and 2 were lost to follow-up. The contralateral testis of the 37 children who underwent orchiectomies developed normally without torsion.

**Conclusions:**

The clinical manifestations of testicular torsion in children are diverse and can easily lead to misdiagnosis. Guardians should be aware of this pathology and seek timely medical attention. When the initial diagnosis and treatment of testicular torsion is difficult, the TWIST score during the physical examination may be useful, especially for patients with intermediate-to-high risk scores. Color Doppler ultrasound can assist in making the diagnosis, but when testicular torsion is highly suspected, routine ultrasound is not necessary as it may lead to delayed surgical treatment.

## 1. Introduction

Testicular torsion (TT), the clockwise or counterclockwise rotation of the testis along the longitudinal axis of the spermatic cord, is a common pediatric urologic emergency that can lead to testicular circulation disorders, testicular ischemic damage, and testicular loss (1). It is generally accepted that torsional necrosis of the testis occurs within several hours of TT (2). Early accurate diagnosis and intervention are extremely important. The typical clinical manifestations of TT are scrotal redness, swelling, and pain on the affected side. However, some children may present with atypical symptoms, such as abdominal pain, nausea, vomiting, groin pain, refusal to feed, irritability, fever, and other non-scrotal symptoms, which may lead to misdiagnosis with gastrointestinal disorders, urinary tract diseases, and appendicitis (3) and delay treatment. The main purpose of this study was to summarize the characteristics and outcomes of children who presented with non-scrotal symptoms of TT at our department. Findings are intended to reinforce the importance of a comprehensive physical examination and ultrasound examination of children with atypical symptoms of TT, which may improve the likelihood of testicular salvage.

## 2. Materials and methods

### 2.1. Patient population

After approval by the ethics board of Anhui Provincial Children’s Hospital, a retrospective review of 73 pediatric patients diagnosed with TT on preoperative ultrasound and surgery was performed. Cases of epididymitis and torsion of the testicular appendages that presented with scrotal swelling were excluded. The age range of included patients was 8 months to 16 years, with an average age of 4.6 years. Patients with perinatal TT were excluded in our study. Left-sided TT was diagnosed in 48 patients, while 25 cases were on the right. The time from the onset of initial symptoms to surgery ranged from 5 h to 7 days, with an average of 2.9 days. Twenty-seven cases had non-scrotal initial symptoms, including 5 with lower abdominal pain, 8 with nausea and vomiting, 3 with periumbilical pain, 3 with crying and refusal to feed, 3 with inguinal pain, 4 with lumbar pain and discomfort, and 1 with a fever. A comparison of characteristics of patients with non-scrotal initial symptoms who were initially misdiagnosed (*n* = 27) with those who had a correct initial diagnosis (*n* = 46 cases) is shown in Table 1.

**Table 1:** Comparison the characteristics of the misdiagnosis and clear diagnosis groups.

Factors	Misdiagnosis group	Diagnosis group	*t*(*x*^2^) value	*P-*value
	(*n*** **=** **27)	(*n*** **=** **46)		
Age at surgery (years)	5.5 ± 1.8	6.2 ± 3.5	1.125	0.235
Affected side			0.016	0.900
Left	18(66.67%)	30(65.22%)		
Right	9(33.33%)	16(34.78%)		
Number of visits				0.000^ab^
≥2 times	13(48.15%)	0(0%)		
1 time	14(51.85%)	46(100%)		
TWIST score(points)			0.730	0.761^a^
Low(0∼2)	1(3.70%)	2(4.35%)		
Intermediate (3∼4)	10(37.04%)	21(45.65%)		
High (5∼7)	16(59.26%)	23(50%)		
Guardian			0.495	0.831^a^
Parents	16(59.26%)	24(52.17%)		
Grandparents	10(37.04%)	20(43.48%)		
Near relation	1(3.70%)	2(4.35%)		

^a^ Fisher’s exact test.

^b^ *P *< 0.05.

As the clinical manifestations of TT vary, making it difficult to distinguish it from diseases with similar symptoms such as epididymitis and appendage torsion, Barbosa (4) developed the TWIST scoring system to assess the risk of TT in children based on their clinical presentation. The scoring system includes testicular swelling (2 points), testicular hardness (2 points), absence of the cremasteric reflex (1 point), nausea/vomiting (1 point), and a high-riding testis (1 point). The total score is used to classify patients into low, intermediate, and high-risk categories (0–2 points, 3–4 points, 5–7 points). The TWIST scores of the children with TT in the present work are summarized in Table 1.

### 2.2. Management and follow-up

**Figure 1:**
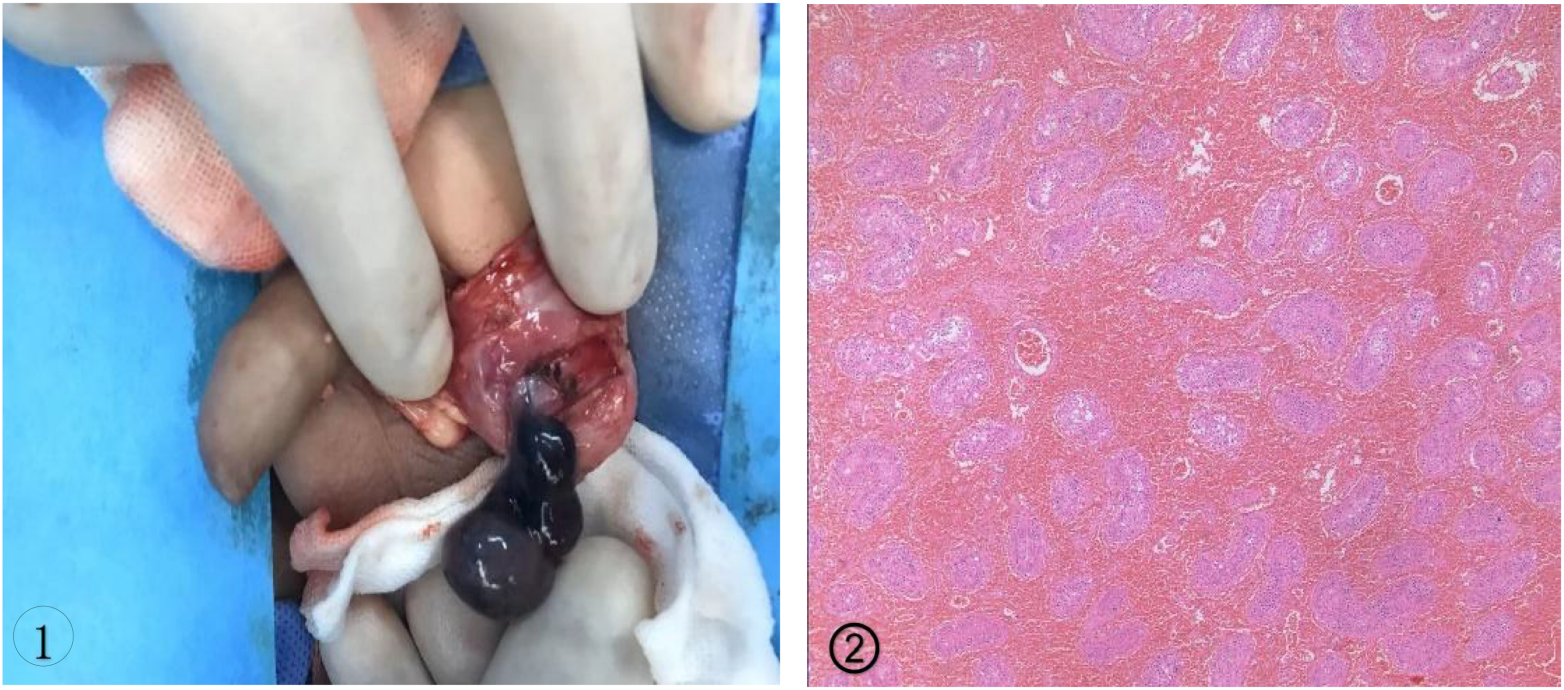
① Tunica vaginalis completely encircled the testis and epididymis was confirmed as bell clapper deformity of the left testis; ②. 2461;. Coagulative necrosis of the seminiferous tubules and interstitial hemorrhage were seen on pathology.

Manual reduction was not performed on any of the cases, and all patients underwent emergency inguinal or scrotal exploration through a longitudinal incision. Some patients had twisted testicles that appeared dark intraoperatively (Figure 1 ①). All were confirmed to have TT. After manual reduction of the twisted testicle intraoperatively, the testicle and spermatic vessels were wrapped in warm saline-soaked gauze and warmed for 20 min to observe testicular blood flow recovery. Bleeding and exudation times were measured after the testicular tunica albuginea was incised to the substance. Grade I was defined as immediate bleeding and exudation, within 10 min as Grade II, and not appearing within 10 min as Grade III (5). Grade I and II cases are candidates for preservation and testicular fixation, while Grade III requires the removal of the twisted and necrotic testicle for pathologic examination (Figure 1②) followed by fixation of the contralateral testicle.

Outpatient follow-up occurred 1, 3, 6, and 12 months post-operatively, testicular palpation and color Doppler ultrasound examinations were conducted to characterize testicular development.

### 2.3. Statistical analysis

All the statistical analyses were performed using SPSS Statistics, version 25.0 (IBM Corp., Armonk, NY, United States). Continuous variables were presented as mean±standard deviation (SD) and categorical variables were presented as ratios(%). Comparisons were performed via chi-squared or Fisher’s exact test for categorical variables. *P* < 0.05 was considered statistically significant.

## 3. Results

Of the 73 patients were included in our study, the degree of torsion ranged from 90° to 960°. The tunica vaginalis completely surrounded the testis, epididymis, and distal spermatic cord rather than connecting to the posterolateral portion of the testis and the base of the epididymis, confirming the bell clapper deformity. As a result, the testis and epididymis hung freely in the tunica vaginalis-created sac (Figure 1①). An orchiectomy without restoration of blood supply was performed in 37 cases while 35 underwent orchiopexy with blood supply recovery. One patient underwent orchiopexy despite the discovery of non-vital testicle intraoperatively at the request of his parents.

Twenty-seven patients were initially misdiagnosed, of which 8 were initially diagnosed as gastroenteritis or mesenteric lymphadenitis at other hospitals and treated with oral antibiotics with poor results. TT was found upon re-examination and emergency surgery was performed. Six patients with transferable right lower abdominal pain around the navel were initially diagnosed with appendicitis, but after ultrasound ruled out appendicitis, TT was confirmed. Four patients with lower back pain were initially diagnosed with urinary tract stones, but after ultrasound ruled out a stone, TT was found. One patient was initially diagnosed with constipation and treated with laxatives, but TT was diagnosed upon arrival at the hospital due to persistent pain. One patient who presented with a lump and pain in his groin was initially diagnosed with an incarcerated hernia with cryptorchidism, but intraoperative confirmation revealed that the testis was pulled and twisted to the groin.

The distribution of TWIST scores within the misdiagnosis group was as follows: 1 low risk (0–2 points), 10 intermediate risk (3–4 points), and 16 high risk (5–7 points). Children with an intermediate-to-high risk of TT accounted for 96.3% (26/27) of the total group. The distribution of TWIST scores within the clear diagnosis group was as follows: 2 low risk (0–2 points), 21 intermediate risk (3–4 points), and 23 with high risk (5–7 points). Children with an intermediate-to-high risk of TT accounted for 95.6% (44/46) of the total group. There was no statistically significant difference in the TWIST scores of the two groups (*P *> 0.05) (Table 1). However, there were significant differences in the time from the onset of initial symptoms to surgery, the total number of patient visits, the degree of TT, and the testicular removal rate between the two groups (*P *< 0.05). Age, affected side, guardian, direction of TT, intra-scrotal or extra-scrotal torsion, and Arda grading were equivalent (*P *> 0.05) (Tables 1, 2).

**Table 2:** Intraoperative findings of the misdiagnosis vs. clear diagnosis groups.

Factors	Misdiagnosis group	Diagnosis group	*t* (*x*^2^) value	*P-*value
	(*n* = 27)	(*n* = 46)		
Initial symptoms to surgery (h)	67.2 ± 33.2	36.5 ± 12.6	5.373	0.000^a^
Direction of torsion			0.517	0.472
Clockwise	17(62.96%)	25(54.35%)		
Counter clockwise	10(37.04%)	21(45.65%)		
Degree of torsion(°)	587.5 ± 155.9	347.8 ± 106.2	3.770	0.026^a^
Type of torsion			0.000	1.000
Intra-vaginal	25(92.59%)	43(93.48%)		
Extra-vaginal	2(7.41%)	3(6.52%)		
Arda classification			5.780	0.056
I	3(11.11%)	11(23.91%)		
II	5(18.52%)	16(34.78%)		
III	19(70.37%)	19(41.31%)		
Testicular treatment			4.378	0.036^a^
Orchiectomy	18(66.67%)	19(41.31%)		
Orchiopexy	9(33.33%)	27(58.69%)		

^a^ *P *< 0.05.

Postoperative follow-up was 6–40 months. One patient of the 36 who underwent an orchiopexy had testicular atrophy after six months (his testis was found to be non-viable during surgery), 2 patients were lost to follow-up, and the remaining 33 did not have testicular atrophy. The contralateral testis of all 37 children who underwent orchiectomies developed normally without torsion.

## 4. Discussion

TT is a common pediatric urologic emergency that often occurs in neonates and adolescents, particularly after intense physical activity or cold weather conditions (2, 6). The definition of bell clapper deformity depends on the posterior tunica vaginalis parietal lamina attachment point,or more exactly, the posterior reflection point of the parietal and visceral layers of the tunica vaginalis (7).A normal testicle has the tunica vaginalis parietal lamina attached to the epididymis posterolaterally and the lower pole of the testis. In patients with the bell clapper deformity, the tunica vaginalis parietal lamina attachment completely encircles and attaches more proximally to the distal spermatic cord. Consequently, up to the point of the tunica vaginalis visceral layer reflection, the testis, epididymis, and distal spermatic cord float freely in the tunica vaginalis (Figure 1①). As a result of the twisting of the distal unattached spermatic cord, the testicle is at danger of intravaginal torsion.

The clinical presentation of TT can vary, but its typical symptoms include redness, swelling, and scrotal pain on the side of the torsion. It can also include nausea, vomiting, abdominal or groin pain, and fever. However, in cases where scrotal symptoms are not the initial manifestations of the torsion or when the torsion is incomplete, symptoms outside of the scrotum may be present. These include abdominal pain, nausea, vomiting, groin pain, fever, or a refusal to eat and irritability in younger children. In these patients, TT can be easily misdiagnosed or missed by the initial treating physician (8). The reasons for these non-scrotal symptoms include the following: (1) The nerves that innervate the testicle and abdomen have overlapping pain distributions. Sympathetic nerve fibers that supply testicular pain originate from T10 to T11, and pain may therefore radiate to the adjacent or same spinal segment that supplies the abdomen. The scrotum is innervated by branches of L1 and S2–S3. Thus, pain may be felt outside the scrotum during the early stages of torsion and scrotal pain may not be evident (910). Additionally, the testicular vessels lie retroperitoneally, so pain can radiate to the abdomen or back along the spermatic cord after torsion (10). (2) TT involves a private area; when adolescents describe symptoms outside the scrotum they may not disclose scrotal pain, leading to misdiagnosis. Younger children may not be able to provide an accurate medical history, leading to delayed treatment (11). (3) The initial doctor may have inadequate experience in the diagnosis and treatment of TT. Due to its non-specific presentation and the difficulty with eliciting accurate information about scrotal pain from children and their parents, TT can be misdiagnosed as appendicitis, gastroenteritis, incarcerated hernia, and urolithiasis (12). Moreover, since most cases of TT occur at night or during the cold season, some initial doctor may not perform a comprehensive physical examination or scrotal ultrasound, which can lead to a missed diagnosis.

At present, the diagnosis of TT mainly relies on a comprehensive physical examination by the initial doctor and a color Doppler ultrasound examination. A sonographer can diagnose TT based on decreased blood flow to the testicle. Although color Doppler ultrasound has a high accuracy, convenient implementation, simple detection, and a low cost, this mode of diagnosis has certain limitations. Color Doppler ultrasound examination has a sensitivity of 69%−91% and a specificity of 87%−100% for the diagnosis of TT (13–15). Ultrasound images typically show decreased or absent testicular arterial blood flow and a swirl sign (sudden change in the course of the spermatic vessels with a helical twist in the outer inguinal ring or scrotum) (16). The early manifestations of TT are occlusion of testicular venous blood flow with continued arterial blood flow (with occasional compensatory increases in blood flow). There are obvious color blood flow signals in the testis parenchyma (17). Early ultrasonography of patients with TT therefore can’t confirm this diagnosis and delays treatment. Further, ultrasound examination results depend on the subjective judgment of the ultrasound equipment and sonographer. An inexperienced sonographer or hospitals that lack ultrasound equipment increase the likelihood of a missed diagnosis. Barbosa (4) proposed in 2013 that the first-visiting doctor perform the TWIST score according to the patient’s clinical symptoms and signs. As the negative and positive predictive values of the TWIST for both low- and high-risk TT are 100%, ultrasonography is not required in either category of patient. High-risk patients should proceed directly to surgery to avoid delaying testicular rescue time with an ultrasound examination. However, ultrasonography is necessary for intermediate risk patients. In this study, of the 27 children in the misdiagnosis group, 96.3% (26/27) were classified as intermediate-to-high risk and 3.7% (1/27) were classified as low risk. Within the diagnosis group, 95.6% (44/46) were classified as intermediate-to-high risk and 4.4% (2/46) were classified as low risk. A common reason for misdiagnosis was that the early symptoms and signs of scrotal pathology were not obvious, leading to an initial diagnosis of gastroenteritis, appendicitis, and mesenteric lymphadenitis. When the child returned to the hospital because their symptoms did not improve, the clinical symptoms of TT were evident, resulting in a higher risk score. In contrast, three children with low risk scores visited due to nausea and vomiting. An ultrasonography revealed reduced blood flow to the testis compared to the contralateral side. A 90-degree TT was found and reduced and blood flow was restored after detorsion, permitting an orchiopexy.

TT can be diagnosed definitively based on its clinical features and ultrasound-assisted examination. As the likelihood of testicular salvage and the length of symptom persistence are inversely proportional in patients with TT, early testicular detorsion and surgical exploration are crucial (18). In cases where immediate surgical treatment is not feasible, manual reduction can be attempted by rotating the right testicle counterclockwise and the left testicle clockwise at an angle standing face-to-face with the patient (19). Patients usually experience immediate relief of pain if manual reduction is successful. However, it should be noted that the testis may be twisted again after successful manual reduction. Therefore, regardless of the success of manual reduction, emergency surgical exploration is still required (20).

In summary, TT is a pediatric surgical emergency with a diverse clinical presentation. It is important to raise the awareness and attention of primary care physicians, emergency physicians, and urologists of the non-scrotal initial symptoms of TT in children. When ultrasound equipment or the experience of ultrasound technicians is limited, the TWIST scoring system can be used as part of a comprehensive physical examination. An intermediate-to-high risk TWIST score plays an important role in indicating TT. Therefore TWIST score is only important for surgeons when have a physical examination but not to those referring to surgery. Any suspicion of TT deserves being examined by pediatric urologist or surgeon. It is very important because of being associated with organ loss even 1 in 30 cases of low risk by scoring. When TT is highly suspected, timely surgical exploration or referral is necessary to avoid the consequences of delayed treatment. In addition, further cases need to be collected to summarise the experience of diagnosis and treatment of TT in children with non-scrotal initial symptoms, due to the small number of cases in this study and the short follow-up period.

## Data Availability

The original contributions presented in the study are included in the article, further inquiries can be directed to the corresponding authors.
